# A critical systematic review of K-12 neurology/neuroscience pipeline programs

**DOI:** 10.3389/fmed.2023.1281578

**Published:** 2023-12-11

**Authors:** Mia T. Minen, Naomi Lebowitz, Jane Ekhtman, Khushalee Oza, Ishah Yusaf, Aarti Katara, Ramisha Aymon, Caitlin Plovnick

**Affiliations:** ^1^Department of Neurology, NYU Langone Health, New York, NY, United States; ^2^Barnard College, Columbia University, New York, NY, United States; ^3^The City College of New York, New York, NY, United States; ^4^Medical Library, NYU Grossman School of Medicine, New York, NY, United States

**Keywords:** K-12, education, neurology, neurology pipeline, neuroscience

## Abstract

**Background:**

Early exposure to neuroscience is imperative to strengthening the neuroscience and neurology pipeline and may present an avenue for increasing the number of practicing neurologists and diversifying the neuroscience workforce. Our objective was to systematically review existing K-12 neuroscience education and outreach programs to understand what educational programs have been developed and implemented.

**Methods:**

We conducted an electronic database search of PubMed, EMBASE, PsycINFO, Education Source, and ERIC. All eligible articles were systematically reviewed to examine the type of program developed, target age group, implementation, and efficacy.

**Results:**

Our search produced 2,574 results, from which 23 articles were deemed eligible. The breakdown by age group was as follows: 5 elementary school, 8 middle school, 8 high school, and 2 general K-12 range of students. Six articles described programs intended for URM students. All programs were found to be successful in exposing students to neuroscience and inspiring interest in pursuing a career in the field of neurology.

**Discussion:**

Further efforts are necessary to analyze the long-term effectiveness of K-12 neuroscience education and outreach programs in overcoming the shortage of neurologists and explore the impact of mentorship for various age groups among K-12.

Systematic review registrationhttps://doi.org/10.17605/OSF.IO/2G8CN.

## 1. Introduction

The shortage of neurologists facing the healthcare field creates an imperative to generate enthusiasm among students for a career in neurology (the branch of medicine concerned with diseases and conditions of the nervous system), neuroscience (the general study of the nervous system), and cognate fields (1). Addressing this shortfall necessitates innovative approaches to garner student interest in the field of neurology early-on in their academic and professional careers. Our strategy is, thus, to investigate early onset neurology education and outreach programs for students in grades K-12. In this context, we define such programs as any initiative designed to expose, introduce, or strengthen knowledge and interest in neurology, as well as careers in neurology and its related fields, such as neuroscience. Existing literature demonstrates a significant connection between a student who studies neuroscience in their undergraduate years, and goes on to pursue either neurology in medical school or a neurology-related career. Complementary to this, more recent research presents the notion that many undergraduates studying the neurosciences yearn for earlier exposure to this intriguing field, as early as the age of thirteen (2–4). Early neuroscience outreach can even leave long-lasting impacts to medical students and neurology experts as well, further highlighting its importance in attracting and retaining student interest within the field. Of important note for gaining historically marginalized student interest in neurology, African American medical students especially attributed inadequate early exposure to neurology to being a major deterrent in pursuing a neurology career, outweighing any other negative impressions of the field (5). Considering this, we set out to critically assess neurology education and outreach programs catered to elementary, middle, and high school students to better understand efforts to introduce students early on to neurology and its related fields.

Early neuroscience education and outreach programs help to cultivate an initial interest among students. This interest in neurology and adjacent fields among students is retained especially when it is augmented with supplementary coursework or extracurricular experience (4). Literature conveying the success of early exposure to Science, Technology, Engineering, and Mathematics (STEM) disciplines to strengthen the STEM pipeline can serve as the groundwork for the implementation of early exposure programs, in a similar fashion, to strengthen the neurology pipeline. Established scientists and engineers frequently attribute interactions with educators and other experts, especially at middle and high school levels, as a positive catalyst for their career trajectories (6–8). Furthermore, scientists have pinpointed the presence of interactive science classrooms, such as those that engage students in hands-on wet-lab work to supplement traditional lecture-style instruction, in middle schools, as a positive influence toward their career choices (9).

Early exposure to neurology and/or neuroscience in K-12 curricula is critical to strengthening the neurology pipeline and increasing the number of practicing neurologists. Furthermore, these programs have the potential to contribute to a more inclusive and diverse landscape within the field of neurology, by welcoming individuals from diverse backgrounds and can offer their distinct viewpoints and experiences. Recent findings by the American Academy of Neurology stressed this disconcerting lack of diversity within the field, with only 2.8% of US neurologists identifying as Black or African American, 7.2% identifying as Hispanic or Latino, and the majority, 68.1%, identifying as White. Moreover, merely 34.7% of neurologists were female (10). Bearing these statistics in mind, it is imperative to utilize the capabilities of neurology education and outreach programs to overcome these gender and racial disparities in neurology by engaging historically marginalized students early on in their K-12 education.

Due to the pressing need for more practicing neurologists and the link between early exposure and pursuing careers in neurology, now is an opportune time to revisit and evaluate existing K-12 neurology and neuroscience pipeline programs. The current academic landscape stresses diversity and inclusion, making it a strategic moment to analyze these programs, their goals, and whether they attract students from various backgrounds and foster this interest in neurology as they aim to do. Additionally, advances in education methodologies call for a thorough evaluation to keep programs aligned with the latest pedagogical approaches in the field. Policymakers, academic committees, and funding agencies are increasingly supportive of STEM education, further underscoring the relevance of this study.

In acknowledging the need to foster early interest in neurology and evaluate K-12 neurology education and outreach programs, we conducted a systematic literature review to assess programs’ goals of attracting curiosity and passion for neurology careers. As far as our knowledge extends, there exists no systematic review that addresses this research question, making this investigation exceptionally valuable in comparison to relying on traditional narrative review alone. The systematic review methodology is an appropriate method of research for this particular study because it provides a structured and transparent approach, enabling a critical examination of all published literature on available programming. While a traditional narrative review may encompass a greater number of informal and ongoing K-12 neurology and neuroscience programs, a systematic review surveys those programs in a more scientific way. By evaluating programs that qualify in a systematic review rather than a more narrative literature review, people will be able to better understand why the particular programs were selected. More specifically, by systematically evaluating the literature on available programs, it is easier to distinguish those programs exclusively designed for K-12 students from programs that encompass a broader demographic or primarily target undergraduate and medical students. Furthermore, the nature of systematic reviews encourages replicability and allows for other researchers to enrich our methodology to evaluate programming that has taken place and been published after our chosen date range, showcasing progress or advancement. Narrative reviews, while informative, may bring forth subjectivity and bias in the selection and interpretation of studies. On the other hand, a systematic review’s structured approach minimizes bias and provides a more objective and rigorous analysis of the existing evidence. This objective lens is crucial when assessing the efficacies of educational programs like K-12 neurology initiatives.

Our study offers a comprehensive analysis of literature on K-12 neurology pipeline programs, that we have identified through systematic search criteria, seeking to gain valuable insights into what factors make them successful in piquing students’ interest in neurology and what areas may need refinement. We specifically address the content and duration of these programs, their methods of efficacy assessment (where applicable), and whether they are designed to engage historically marginalized audiences. Furthermore, where relevant, we provide a comparative analysis of programs targeting different age groups, including elementary, middle, and high school students, based on the same criteria. By employing these systematic review techniques, our research offers a more robust, rigorous and forward-looking approach to evaluating K-12 neurology education and outreach efforts. While K-12 neurology pipeline programs are scarce, this underscores our ultimate goal through this review: by showcasing the existing literature that is available, we hope this serves as a promotion for the development and implementation of programs that foster more K-12 student in neurology.

## 2. Methods

We developed a systematic literature search to uncover research on programs related to neurology and/or neuroscience education and outreach. To do so, we formulated the following question using the Population, Intervention, Control, and Outcome (PICO) framework: What programs in neuroscience education have been developed and implemented to pique the interest of elementary, middle, and high school students? We focused our population for this systematic review on K-12 students; studies of programs that focused on populations of undergraduate students were evaluated in a separate review (In Press). Interventions referred to neurology/neuroscience education and outreach programs, defined as any initiative aimed at exposing, introducing, or strengthening students’ knowledge and interest in neurology or neuroscience, and inspiring them to consider pursuing a career in this field. We measured the outcomes by assessing how these programs were implemented, to whom they were delivered (including any programs designed specifically for historically marginalized students, given the shortage of neurologists from these groups), and whether any guidelines were utilized to evaluate their efficacy (i.e., to what extent these programs claimed to have achieved their stated goals).

This study was pre-registered on July 21st, 2022, via Open Science Framework (OSF) Registries. A team of six undergraduate research assistants (NL, JE, KO, IY, AK, and RA), under the guidance of a neurologist (MTM), developed a list of keywords related to neurology and neuroscience education and career pipelines, which a medical librarian (CP) then expanded and refined. The librarian searched PubMed, EMBASE, and PsycINFO via the Ovid platform and Education Source and ERIC via the EBSCO platform for articles describing neuroscience and neurology education and outreach programs. For our study, programs were included if they exposed, introduced, or strengthened knowledge and/or interest in neurology or a related field, including neuroscience. Each search strategy had a combination of keywords and controlled vocabulary appropriate to each database. The complete search details can be found in Appendix A.

**Figure 1:**
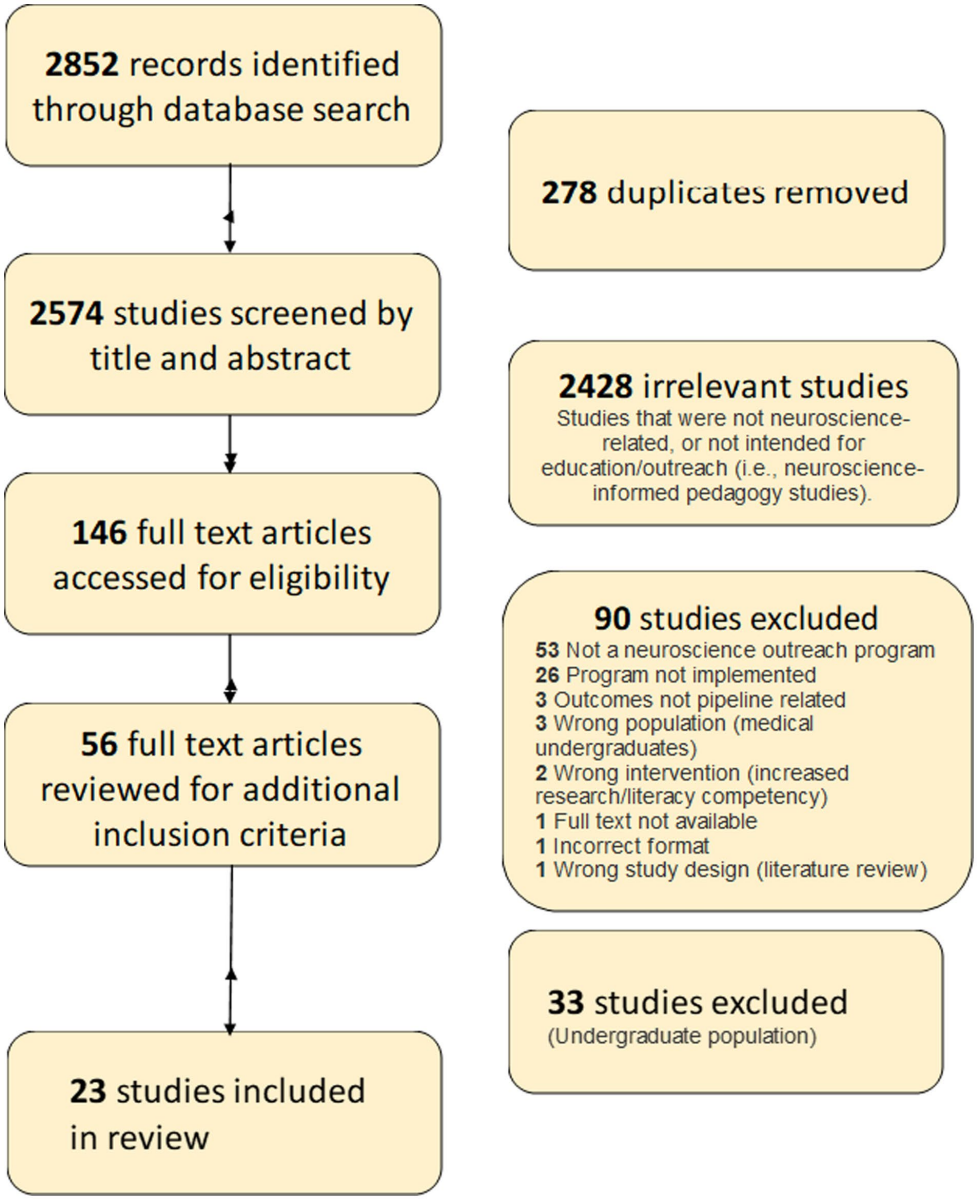
PRISMA diagram.

The search was conducted on July 5, 2022, and was not limited by language or publication date. Resulting citations and abstracts were imported into Covidence software for screening. Figure 1 illustrates our search process via a PRISMA (Preferred Reporting Items for Systematic Reviews and Meta-Analyses) diagram. Beginning with 2,852 studies, 278 duplicates were removed, and 2,574 studies were screened by the team of reviewers (NL, JE, KO, IY, AK, and RA) based on the following inclusion criteria: (1) the program had to be related to neuroscience or neurology, (2) the program needed to be already implemented and provided specific outcomes, and (3) the population of program participants was limited to students in K-12 education or undergraduate college students. Before Covidence screening, all reviewers met with the medical librarian to review the screening process and ensure consistency in technique. Each citation was independently screened by two different reviewers and received two votes, after which disagreements were resolved through discussion resulting in a consensus. In total, 146 studies were selected for full-text review and were screened by the same reviewers, following the same process of two independent reviewers per article. Reviewers only included studies for full-text review if they discussed implemented programs, rather than theoretical or in the lesson plan format.

Fifty-six studies passed the inclusion criteria and underwent a second round of full-text review, to separate studies based on program participants, K-12 students and undergraduate college students. Of these 56 articles, 20 were relevant to the K-12 age group (s), 28 were relevant to undergraduates, and 8 were programs that were relevant to both undergraduates and one or more K-12 groups. In the mixed group, reviewers evaluated the programs’ goals from each study to determine which age groups were benefitted more directly. Studies geared exclusively toward a K-12 group rather than an undergraduate group were included in this systematic review and marked with a double asterisk (**) in Table 1. Only studies from our original search were included in this systematic review. Twenty-three studies were ultimately included for this review, 20 specifically targeted to K-12 students, and 3 from the mixed group of studies. This systematic review process was concluded in August of 2022 and analysis began afterwards.

**Table 1:** Description of studies meeting eligibility for the systematic review – elementary school.

First Author/Year/Journal/Title	Type of program (**)	Goal of program (*)	Developed by/participants	Program delivery	How was program studied	Program outcomes
(Fitzakerley, 2008) *Minnesota Medicine* Service learning in rural communities. Medical students teach children about the brain	Medical student community service requirement for teaching elementary school students about the brain during “Brain Awareness” sessions.	Increase elementary students’ knowledge of the brain, inspire young children to pursue science and health care.	Developed by university faculty. Participants: more than 10,000 elementary school students.	Presentations to classes take 45 min-1 h, where each medical student presents to around 3 classes.	Elementary students reflected on their experiences, informally.	Served as a resource for elementary students and teachers.
(Fitzakerley, 2013) *PLOS ONE* Neuroscientists’ classroom visits positively impact student attitudes	University-school partnership featuring scientist-in-the-classroom visits from faculty, staff, and students of university to elementary students during “Brain Awareness” sessions.	Increase students’ appreciation of their own ability to learn and contribute to their general understanding of basic brain function.	Developed by neuroscientists. Participants: 106 classrooms reached in present paper, but more classrooms since 1990s.	Neuroscientists and students from university campuses presented to classes for 45 min-1 h, including interactive activities about the brain and real human/animal brains.	Pre/post-survey for students included a Likert scale (18 forced-choice items on attitudes toward science and own ability to learn) and open-ended questions. A teacher survey rated value of presentations on Likert scale and with two open-ended questions.	Pre- to post-survey change showed sessions increased positive attitudes toward science and growth mindset in students. >95% of teachers said that the visits stimulated students’ interest in the brain.
(Mohd Ibrahim, 2015) *Malaysian Journal of Medical Sciences*Neuroscience Club in SKKK3 and SMSTMFP: The Brain Apprentice Project	Implementation of Neuroscience Club as part of school curricula with neuroscience activities and participation in brain competitions.	Promote science and the neurosciences beyond conventional classroom teachings and assist in the delivery of neuroscience knowledge as part of the cultivation of neuroscience as a career option.	Developed by graduate interns/neuroscientists. Participants: 42 members in one school club and 80 members in another school club.	Over 12 months, neuroscience clubs incorporated school-led activities and graduate intern-led neuroscience activities in two phases.	Students shared experiences anecdotally, as did their teachers.	Club members showed great interest in all of the club’s activities and their performance on the Primary School Achievement Test and Malaysian Certificate of Education examinations improved tremendously.
(Bazzett, 2018) *JUNE* Engaging, Entertaining, and Educating Underserved and At-Risk Youth with STEM-Based Activities	University students majoring in neuroscience engage in service-learning by teaching underserved and at-risk 4-6-grade students about STEM concepts, specifically neuroscience, and serve as mentors for the children.	Increase at-risk and under-served 4-6-grade students’ interest in neuroscience and expose them to broader STEM-related careers, using undergraduate students majoring in neuroscience as instructors/mentors. *	Developed by neuroscientists and faculty. Participants: 10–12 students take part in program each semester, with capacity for 20 students max.	1.5-h long afterschool program 3 days a week during the school year (neuroscience-specific content is offered only 1 day a week) were delivered. Undergraduate instructors presented highly interactive lessons and hands-on neuroscience experiments and demonstrations for the students. Some lessons included trips to college laboratories.	Effectiveness of the program measured by changes in students’ level of interest in STEM. Rudimentary assessment of student neuroscience learning was conducted using bingo-like game.	No numerical data regarding measures of student interest in STEM. Anecdotal reports from undergraduate instructors/mentors indicate the program is worthwhile for elementary-school students.
(Toledo, 2020) *JUNE* Interactive Student-Centered Neuroscience Workshops for Sixth Graders Enhance Science Knowledge and Education Attitudes	Scientist-in-the-classroom visits from undergrads to 6th graders to deliver interactive neuroscience workshops.	Improve students’ neuroscience knowledge and education attitudes in socioeconomically disadvantaged areas.*	Developed by undergrads and supervised by faculty. Participants: 77 sixth graders.	Students attended 5 h-long workshops over five consecutive weeks. Workshops included interactive brain models, construction of a neuron, and simplified lessons.	Pre- and post-test on education attitudes administered, as well as multiple choice test on neuroscience concepts.	Paired sample *t*-test showed significant improvement in 7 out of 8 neuroscience knowledge areas and 4 out of 6 educational attitude items.

## 3. Results

### 3.1. Publications analysis

The articles were published between 1999 and 2021, with 18/23 (78.2%) published in the last 10 years. The age range varied, with 5/23 (21.7%) of the K-12 group articles describing programs for elementary school students, 8/23 (34.8%) describing programs for middle school students, 8/23 (34.8%) describing programs for high school students, and 2/23 (8.7%) describing programs for K-12 students. Only 6/23 (26.1%) of the articles explicitly referenced a historically marginalized demographic, with an even breakdown of elementary (11, 12), middle (13, 14), and high school groups (15, 16). Programs which specifically engaged historically marginalized students are marked with an asterisk (*) in Table 1. About 40% (9/23) of the articles were published in neuroscience journals (11, 12, 14, 15, 17–21) and four were published in medical journals (13, 22–24).

### 3.2. Content analysis

Every program was developed in partnership with or received input from neuroscientists and/or faculty at universities or research institutions. As a result of a collaboration with community institutions, interventions were delivered to students limited to certain school districts or localities. Although many students within a particular area could participate in a program, programs were only available to a small, cross-country population (Table 2).

**Table 2:** Middle school.

First Author/Year/Journal/Title	Type of program (**)	Goal of program (*)	Developed by/participants	Program delivery	How was program studied	Program outcomes
(Cunningham, 1999) *Academic Medicine* The University of Washington and partners’ program to teach middle school students about neuroscience and science careers	Brain Power Van with science educators and 33 interactive exhibits on neuroscience and health careers visits middle schools for assemblies. Summer Institute educates K-12 science teachers on neuroscience concepts.	Support science teaching of concepts related to the brain and neurology, enhance K-12 students’ understanding of neuroscience and the use of animals in biomedical research, and promote middle-school students’ interest in scientific careers, especially URM groups (African American, Hispanic, and female students).*	Developed by research scientists and health care professionals. Participants: Reaches 30–35 schools >1,000 K-12 students, 80 teachers each year, additional 30–40 teachers each summer.	Brain Power Van visits each year for special assemblies. Summer Institute trains and summer speakers’ bureau events are scheduled engagements.	Various assessments throughout the years; 1993 and 1997, 14-item multiple choice questionnaire was administered to 100 students each in intervention or control arm; in 1997, a survey on attitudes toward careers in science/health of 178 students who listened to presentations by program speakers.	In both 1993 and 1997, intervention group scores were significantly higher than control group scores on Brainy Questions questionnaire (1997: *t* = −9.5; *p* < 0.000). In 1995 post-survey of Summer Institute teacher participants, 79% (*n* = 30) said they had implemented or plan to implement material they learned into their classroom curricula.
(Miller, 2002) *The Neuroscientist* Teaching Neuroscience through Web Adventures: Adolescents Reconstruct the History and Science of Opioids	Episodic web-based adventure series delivering interactive neuroscience-based lessons on the science and history of opioids to middle school students.	Engage middle school students in neuroscience education through the Internet.	Developed by neuroscience researchers, science teachers, clinicians, middle school student and parent advisory boards. Participants: 148 (88 girls, 60 boys).	Two of four episodes (~20 min each) were evaluated in three middle schools (varying demographics reported in article). Science classes of ~25–30 seventh graders independently completed the episodes in their school computer labs.	Without any introduction or background lessons, researchers administered to students pre-test ≥2 days before intervention, opinion questionnaire immediately after, and post-test ≥2 days after intervention. Both episodes were evaluated together.	Paired *t*-tests found significant increases in test scores from pre to post-tests in all classes. Specific concepts were matched as much as possible between pre- and post-test—questions were asked in alternative forms covering same concepts and enabled comparison of students’ knowledge gained in specific areas of content.
(Miller, 2006) *CBE – Life Sciences Education* An Online, Interactive Approach to Teaching Neuroscience to Adolescents	Episodic web-based adventure series delivering interactive neuroscience-based lessons on “club drugs” and drug abuse to middle school students.	Engage middle school students in neuroscience education on drug abuse through an active internet experience.	Developed by researchers. Participants: 289 students in seventh and eighth-grade science classes across five schools around the country.	For evaluation in schools, students independently completed the three episodes in the web-based series (~20–30 min each). The series is now publicly available online at no cost.	Without any introduction or background lessons, students were given a 35-item pre-test; 3 days later, students completed the web-based series. After another 3 days, students took a post-test with the same 35 items.	Scores from tests were corrected for guessing. Paired *t*-tests found significant increase in students’ scores on neuroscience content from all three episodes and in all five schools after completing the series.
(MacNabb, 2006) *CBE – Life Sciences Education* Neuroscience in Middle Schools: A Professional Development and Resource Program that Models Inquiry-Based Strategies and Engages Teachers in Classroom Implementation	Brain Science on the Move program consisting of five components: BrainU summer professional development program for teachers to gain neuroscience knowledge and confidence in teaching/incorporating neuroscience into their curricula, Explain Your Brain Assembly multimedia presentation to students, Explain Your Brain Exhibit Stations—interactive table-top activities for students, in-depth class activities and experiments to be delivered by teachers, Brain Trunks materials/resources sets loaned to classrooms.	Enhance middle-school students’ knowledge of and interest in neuroscience. Improve middle-school teachers’ confidence in teaching neuroscience concepts. Program aimed at underserved schools (schools with large minority and free and reduced lunch populations, rural schools).	Developed by faculty, researchers, educators from Science Museum of Minnesota. Participants: over three BrainU cohorts, 56 teachers, 9,023 students.	Teachers participate in 2-week long BrainU during summer, planning implementation of content and activities for upcoming school year. During school year, schools were given 50-min interactive Explain Your Brain assembly (delivered to audiences as large as 300 students) and Explain Your Brain exhibit stations on a different day. Lasted ~1–2 lessons. Brain Trunks were loaned to teachers’ classrooms for 2–3 week increments and came with instructional guides for teachers.	Teacher neuroscience content was evaluated before and after participation in Brain using short multiple-choice test. Teacher confidence in knowledge of and perceived ability to teach neuroscience content was evaluated on self-efficacy scale and value of program was measured using Likert scale in pre- and post-BrainU surveys. After implementing their “action plans,” participating teachers and students were surveyed on overall impact of program.	Student and teacher responses on post-program implementation survey were uniformly positive; teacher responses indicated the Explain Your Brain program and associated activities were valuable to students and teachers, teachers also reported improvement in their students’ understanding of neuroscience concepts and believed both teacher and student outcomes for the program were achieved. Of 2,519 students and 39 teachers across 36 schools participating in the program between 2001 and 2003, 67% of students believed the program was “worthwhile” and 29% believed it was “somewhat worthwhile.”
(Koizumi, 2013) *Neuroscience Research* The Muscle Sensor for on-site neuroscience lectures to pave the way for a better understanding of brain–machine-interface research.	On-site interactive neuroscience lectures using simplified electromyography (Muscle Sensor) device for junior high-school (middle school) students in Japan; lectures consist of background/introduction to the brain, explanation of neurons and electrical signaling, hands-on demonstration of electrical signaling using Muscle Sensor, and introduction to the latest brain-machine interface research.	Engage students in neuroscience learning beyond textbook readings by involving them in hands-on neuroscience demonstrations neural bioelectric signaling.	Developed by researchers. Participants: 100 students surveyed across 19 schools.	Researchers travel to various schools with Muscle Sensor device to deliver 45-min lectures to students. Time during lectures is devoted to student-directed use and exploration of bioelectric signaling using Muscle Sensor. Lectures were given at 19 schools form 2009–2011.	100 students from one of the schools given a lecture were administered a two-item questionnaire both after learning the neuroscience concepts covered in the lecture from their textbooks and after participating in the lecture.	Significant increase in students’ performance on the questionnaire after participating in the lecture.
(Louw, 2018) *Physiotherapy Theory and Practice* Can pain beliefs change in middle school students? A study of the effectiveness of pain neuroscience education	An abbreviated pain neuroscience education lecture to middle school children in PowerPoint form. Themes included a discussion of peripheral sensitization, central sensitization, bio-psycho-social factors associated with pain, and threat appraisal of the brain.	To cause a positive shift in pain knowledge as well as healthier beliefs regarding pain in middle school students.	Developed by neuroscientists. Participants: 133 students.	Students attended a one-time 30-min lecture on pain neuroscience.	Pre- and post-test measures of pain knowledge (neurophysiology of pain questionnaire [NPQ]) and beliefs regarding pain (numeric rating scale) were administered.	Significant improvement in knowledge was found on NPQ test scores. Significant shifts in beliefs were also found in all but one of the pain beliefs questions. Overall, the lecture resulted in a significant increase in their knowledge of pain as well various beliefs regarding pain.
(Vollbrecht, 2019) *JUNE* An Effective Model for Engaging Faculty and Undergraduate Students in Neuroscience Outreach with Middle Schoolers	A course consisting of different activities and presentations for undergrad students to teach middle schoolers. **	This process helped to solidify concepts learned in class and build confidence in their knowledge.	Developed by Undergraduates and faculty. Participants: 174 students from grades 6–8 participated in activities and 22 undergrad students participated as instructors.	It was a course consisting of both presentation and hands-on learning as well. These activities were strategically placed in the same lesson in order to emphasize our primary objectives for students to understand the important role that sensory receptors play in our nervous system.	Outcomes based on the number of correct answers in a questionnaire regarding topics learned during the intervention. Questionnaire was given as a pre-and post-test.	Looking at student performance on an individual level, 68% of students demonstrated an improvement in their post-event assessment, and another 20% maintained the same score.
(Miranda Feitosa, 2021) *Journal of Neuroscience Research* Open Practical Laboratories in the Neurosciences: An outreach program for neuroscience communication in middle schools	Scientist-in-the-classroom outreach program consisting of practical and demonstration activities on the theme of the neurosciences.	To improve the knowledge of the neurosciences by elementary school students in low performance schools and to promote better attitudes in relation to neuroscience, science in general, and scientists.*	Developed by neuroscientists. Participants: 166 students in experimental group, 138 in control group.	Neuroscientists gave oral and demonstrative presentations with a talk, models, and a board game (~1 h and 45 min total).	Pre- and post-test surveys for both experimental and control groups administered, including one neuroscience attitude questionnaire with Likert scale and one neuromyths questionnaire on general knowledge and brain myths.	Based on pre- and post-test comparison, after the intervention, attitudes toward science were more positive with more students stating that they want to be scientists in the future. Also, the proportion of correct answers relating to neuromyths increased significantly when compared to answers before intervention.

Elementary and middle school programs tended to be interactive; for example, undergraduates teaching the after-school neuroscience education program for fourth-six grade students effectively used physical models of the brain and implemented hands-on activities like building edible brains (11). The multifaceted Brain Science on the Move program for middle school students captivates short attention spans with in-class activities such as sheep-brain dissections, investigations of perception using prism glasses, and kinesthetic activities demonstrating neural transmission (13).

On the other hand, programs geared toward high school students tended to be more in-depth in specific areas of neuroscience, like neural communication and computational and developmental neuroscience, with some exceptions, such as the summer neuroscience academy described by Colpitts et al. (15), which provided students with a broader overview of the field and neurology careers. Generally, high school programs focused on more intense exposure to neuroscience and neurology—often tasking students with developing research under the guidance of neuroscience faculty or other professionals or immersing students in-depth to special topics in neuroscience such as neurogenetics and computational neuroscience—rather than generating broad enthusiasm for the field and STEM-related careers, which was more often a goal of elementary and middle school programs (Table 3).

**Table 3:** High school.

First Author/Year/Journal/Title	Type of program (**)	Goal of program (*)	Developed by/Participants	Program delivery	How was program studied	Program outcomes
(Marzullo, 2012) *PLOS ONE* The SpikerBox: a low cost, open-source bioamplifier for increasing public participation in neuroscience inquiry	Open-source tool developed to assist students in easy experiments to help them amplify and be able to listen to the electrical activity of neurons, teachers need minimal training to use said tool.	Help students hear and see activity in neurons and see how other factors impact neural activity, overall engage K-12 students in basic neuroscience education.	Developed by neuroscientists. Participants: students in 2 high school lecture classes, amount not specified.	Classroom workshops 3–6 h long conducted. Between lectures, students assembled their own Speaker Boxes.	Researchers administered pre- and post- workshop surveys to students. 4 separate experiments were conducted, survey results were evaluated.	Pre-to-post survey results showed workshops increased students’ knowledge of both electronics and neuroscience. Positive reactions from students regarding the workshops.
(Shannon, 2014) Portable conduction velocity experiments using earthworms for the college and high school neuroscience teaching laboratory	Portable and robust experimental setup using earthworms that allows students to perform conduction velocity measurement in laboratory session.**	To help high school students and above in increasing their knowledge of neural communication, conduction velocity, and cable theory with hands-on learning.	Developed by neuroscientists. Participants: 15 respondents in college +25 respondents in high school.	Held workshops in a neuroscience class at an undergrad college and a biology class at a high school. 2-h workshops were a mixture of lectures and demonstrations. Students observed and assisted in live demos and engaged in discussion about the theory and experiments.	Before and after the workshops, students at both schools were given multiple-choice tests to examine their knowledge on conduction velocity concepts. High school version did not involve mathematical concepts with time constraint or length constant knowledge.	25% increase in test scores (mean 5.6 for before and mean 8.8 for after) in college students. 11% increase in test scores (mean 2.95 before and mean 3.81 after) for high schoolers. Students increased their knowledge relating to earthworm anatomy and conduction velocity theory but not in nodes of Ranvier or sparse coding for high schoolers.
(Crusio, 2017) *F1000 Research* Engaging high school students in neuroscience research -through an e-internship program	E-internship summer program for high schoolers including behavioral neuroscience, brain disorders, and a research project.	To show that an e-internship program is an effective way to introduce students to advanced neuroscience topics and gain hands on experience.	Developed by neuroscientists. Participants: 15 high school students.	Students offered an internship using open access tools found online and following a laboratory’s protocol for profiling gene expression data. Students were guided through the development of a research project over 6–8 weeks for 10–15 h per week, working asynchronously and in groups. Included access to scientific literature, mentoring, and communication with scientists.	Students completed projects as well as a quantitative and open-ended survey upon completion of the course on internship content, instruction, and overall experience.	Students had positive attitudes toward the course and were interested in their experience of collaborating with scientists on research projects in neuroscience related topics. Outcomes relied only on a self-reported survey with no clear analysis of data.
(Babinski, 2018) *Journal of Adolescent Health* Impact of a Neuroscience-Based Health Education Course on High School Students’ Health Knowledge, Beliefs, and Behaviors	A high school course integrating standard health education with neuroscience concepts in teaching relevance to students’ everyday lives.	Assess the feasibility of integrating neuroscience into health education and observe for students’ interest, knowledge, and self-efficacy surrounding neuroscience and health.	Developed by neuroscientists. Participants: 13 teachers from two high schools, 395 students.	The course was administered in the fall semester for 90 days, with participating teachers also completing a 3-day training.	The students completed online surveys before and after taking the course that assessed knowledge and beliefs on the curriculum material, and teacher interviews were also done for feedback.	Of the 81% of students who answered the most important thing they learned, 37% students in the neuroscience group were more likely to mention the brain. This group also showed increased knowledge in neuroscience concepts.
(Imondi, 2019) *Journal of STEM Outreach* NeuroLab Research Experiences: Extending the CURE Design Framework into an Informal Science Setting Dedicated to Pre-College STEM Instruction	Course-based undergraduate research experience (CURE) adapted for upper-level high-school students; immersive residential summer research and learning experience at multiple institutions.	Increase high-school students’ access to and understanding of basic life-sciences (developmental neuroscience) research using an adapted CURE framework.*	Developed by neuroscientists. Participants: 58 students.	10-day research and learning-intensive institute during the summer. Students rotate through different tasks in their labs, engage in group discussion and individual learning, and hear from guest speakers.	Program assessed by independent educational evaluation firm—students complete surveys on first and last day of program and at one and six-months post-program assessing their understanding of developmental neuroscience concepts and self-efficacy for and attitudes toward conducting research.	Significant increase in students’ efficacy in research and moderate to large gains in collaborative skills. One-month follow-up surveys indicate students perceive their research as valuable to the scientific community. Six-month follow-up surveys indicate students remain aware of the numerous opportunities for continued research and discovery.
(Colpitts, 2019) *JUNE* Development of an Introductory Neuroscience Teaching Experience for Undergraduates with a Low-Cost Neuroscience Summer Academy	Introductory neuroscience academy taught by undergrads.	Provide high school students with an affordable and engaging introductory neuroscience experience *	Developed by undergrads under advisement of a university faculty member with a neuroscience specialty. Participants: 8 high school students.	In summer, students attend four consecutive days of “Brain Camp,” during which they engage in interactive neuroscience learning directed by undergrads.	Undergrad teachers wrote reflections on their experiences teaching and were surveyed regarding their experience with the program.	Based on qualitative observations, high school students were introduced to the field of neuroscience.
(Harris, 2020) *Frontiers in Neurorobotics* Neurorobotics Workshop for High School Students Promotes Competence and Confidence in Computational Neuroscience	1-week introductory neurorobotics workshop for high-school students to teach principles of neuroscience and computational neuroscience.	Enhance high-school students’ understanding of computational neuroscience through hands-on neurorobotics activities.	Developed by neuroscientists, engineers, and educators. Participants: 295 students across two high schools.	Students at each school were divided into 30-person classes and attended 4–5 days of hour-long neurorobotics workshops. Small groups within each class were tasked with designing their own “brain” capable of performing behaviors of the students’ choosing and presenting their ‘brain” to the class.	Pre- and post- surveys with four open-ended questions assessing neuroscience content learning and 14 Likert scale questions assessing attitudes toward science. Due to time constraints on the final day of the workshop, not all students completed the post-survey.	Significant improvement on all neuroscience content questions and in students’ attitudes regarding neuroscience, but not their attitudes toward science in general.
(Frey, 2021) *Medical Science Educator* Impact of Early Introduction to the Neurosciences on West Virginia High School Students via the Brain Bee	Brain Bee—a worldwide non-profit neuroscience education outreach organization which sponsors regional competitions and activity days for high school students.	Garner interest in neuroscience among high school students and inspire future leaders in neuroscience.	Developed by neuroscientists, WV regional Brain Bee is run by medical students and neurology residents. Participants: 34 high school students, majority in 11th or 12th grade.	Students given 3 months to prepare for exams based on neuroanatomy, neuroscience facts, and mock-clinical evaluations. Regional competition lasts 1 day and consists of exams and educational activities (e.g., research lab tours, experimental demonstrations, interactive lectures, and career panels).	Students completed pre- and post-surveys gauging their interest in science and neuroscience and confidence in their own knowledge; free-response answers from students about their experiences were analyzed qualitatively.	Students’ interest in pursuing a neuroscience career and confidence in neuroscience knowledge increased significantly. 96.8% of participants would recommend this program to peers or participate in similar programs in the future. Free responses emphasized the importance of role models, supportive learning environments, and accessibility of information to neuroscience learners of all interest levels.

Of note are the creative physical and conceptual sources such as Backyard Brains that are used by programs to become more interactive and accessible. One example is the Spikerbox, an open-source, low-cost tool to amplify the electrical activity of neurons, which is easy for teachers to master and thus has the potential to serve classrooms even when a neuroscientist is not present (25). Additionally, two programs conducted their work under the title of “Brain Awareness Sessions” suggesting branding of activities with well-known initiatives such as the Dana Foundation’s Brain Awareness Week, and Brain Facts website (22, 26, 27).

Only two of the programs included an explicit mention of a long-term mentorship component (11, 28), although 7 of the remaining programs facilitated an interaction between K-12 students and a neuroscientist (13, 14, 16–18, 29, 30), 3 with a neuroscience undergraduate student (12, 15, 21), 1 with neuroscientists and undergraduates (26), and 3 with neurology graduate/medical students or resident (22–24).

### 3.3. Programmatic run

The number of K-12 participants in each program varied greatly, ranging from as little as eight participants (15) to over ten thousand (22). Out of 23 programs, 3/23 (13%) did not specify the exact number of participants (11, 25, 26), 4/23 (17.4%) had under fifty participants (15, 23, 28, 30), and 3/23 (13%) had between fifty and one hundred participants (12, 16, 18). For the remainder of the programs, 4/23 (17.4%) had between one and two hundred participants (19, 21, 24, 29), 4/23 (17.4%) had between two and four hundred participants (14, 17, 31, 32), 1/23 (4.35%) had over one thousand participants (13), and 2/23 (8.7%) had close to or over ten thousand participants (22, 33).

The duration and length of the programs varied. Twelve programs were brief, lasting 2 h or less, and/or delivered only once (13, 14, 18–22, 26, 29–31, 34), whereas eleven were longer-form content that required multiple days or weeks to implement (11, 12, 15–17, 23, 24, 28, 32, 33). Of the shorter programs, components often included presentations/lectures, interactive activities, and demonstrations. The longer-form programs demanded higher levels of commitment, such as preparation for a brain knowledge competition, creation of culminating research projects, group discussions, online tools, asynchronous work, and field trips.

### 3.4. Programmatic assessment

Two of the programs were analyzed using only qualitative methods (22, 32), fourteen were analyzed using only quantitative methods (12–14, 16, 18–21, 23, 25, 29–31, 33), four of the programs were analyzed using a mixed methods approach (17, 26, 28, 34), and three of the programs used anecdotal evidence (informal reactions from students after the intervention and instructors’ subjective impressions) (11, 15, 24).

All 23 programs reported positive results post-intervention. For the two qualitatively analyzed programs, students completed reflections and answered open-ended survey questions, finding the programs helpful and piquing their interest in the brain (22, 32). All fourteen programs that assessed student learning quantitatively found increased student test scores following participation in the various programs (12–14, 16, 18–21, 23, 25, 29–31, 33). The four papers utilizing a mixed methods approach used a combination of quantitative surveys (usually on a Likert scale) and open-ended questions (15, 17, 26, 34). Likert scales showed more positive attitudes toward science education post-intervention, and open-ended questions showed improvement of neuroscience content knowledge. The three programs, which relied only on anecdotal evidence for efficacy evaluation, found that their respective K-12 groups met their stated goals of providing an introduction to neuroscience topics, and students reported positive feelings toward the topics (11, 15, 24).

### 3.5. Historically marginalized students focus

Of the six papers that referenced a historically marginalized audience of (11–16), two papers engaged African American, Hispanic, and/or female students (13, 16), one paper engaged low-performing schools (14), one paper engaged students attending school in a socioeconomically disadvantaged area (12), and two papers engaged underserved and low-income students (11, 15). The outcomes of all six programs were positive. From pre-to post-test surveys, attitudes toward science were more positive after intervention (14) and increased interest in learning about the brain (13). Programs were delivered in a variety of formats. Both historically marginalized audience programs administered to high school students consisted of summer camp-style programs with rotating neuroscience-related activities (15, 16). One program intended for middle school students was a one-time oral and demonstrative presentation; the other was a Brain Power van visiting students for special assemblies (13, 14). For elementary students, both programs consisted of workshops throughout the day over a few weeks with interactive lesson plans (11, 12).

## 4. Discussion

We believe this is the first systematic review of K-12 programs, specifically assessing how neurology and neuroscience programs may be developed and implemented. The insights from this review hold paramount importance toward forming new initiatives to heighten interest in neuroscience and bolster the neurology pipeline. Recent literature has demonstrated a notable increase in scholarly attention in the field, with more than three-quarters of the papers published in the past 10 years (6–8, 10–12, 15–17, 20, 21, 23, 24, 26, 28–30, 32), contributing to this discourse. Several recurring themes emerged, namely, the involvement of established neuroscientists and/or faculty across universities and research institutions now participating in at least one stage of the development and implementation of neurology education and outreach programs. Equally notably, close to a third of such programs were founded in partnership between universities and K-12 schools (11–13, 15, 16, 21, 22, 26), and the remaining two thirds relied on support from various public resources, ranging from museums, to non-profit neuroscience outreach organizations, to neuroscientists’ laboratories, and interactive online programs developed by neuroscientists. This dynamic collaboration reinforces the need for fostering a mutually beneficial relationship between K-12 schools and teachers and local universities and organizations in the creation of these programs. Strengthening these partnerships might help to provide relevant neuroscience training to students, and working with local organizations could bolster the longevity and feasibility of these programs. However, while these collaborations could prove successful, it would also be good to ensure scalability so that such programs could serve as models that guide future endeavors, e.g., collaborations with national organizations, such as the American Academy of Neurology (AAN), American Neurological Association (ANA) or the National Institutes of Health (NIH), groups who are capable of attracting larger and more diverse audiences, compared to those in this review.

Programmatic aspects and components exhibited considerable diversity. Notably, every program we examined consistently reported positive changes in students’ attitudes toward neuroscience, their knowledge in the brain, interest in STEM careers, and even improvement in exam performance. For elementary students, the engagement primarily revolved around scientist-in-classroom visits from undergraduates, medical students, and faculty (11, 12, 22, 26), alongside participation in school-led activities and competition in an extended afterschool club spanning 12-months (24). Notably, the scientist-in-classroom visits varied, ranging from one-time hour-long sessions on the brain (22, 26) to comprehensive STEM programs with 1.5-h visits 3 days a week (11). The assessment of program outcomes for elementary students predominantly relied on anecdotal feedback from students and staff (11, 22, 24), although some programs did evaluate changes in attitudes toward and knowledge related to science and education (12, 26). For middle school students, the programs encompassed a wide spectrum of approaches, from an asynchronous 1.5-h-long episodic web-based series delivering neuroscience lessons (19, 31), a mobile Brain Van with science educators and exhibits visiting for assemblies (13), Brain Trunks on loan to classrooms with resources (33), and interactive 30 to 45-min lectures (18, 29). A few of these programs employed surveys to gauge shifts in attitudes toward science and neuroscience career interests (13, 33), but the majority administrated pre- and post-tests to assess changes in content knowledge (14, 18, 19, 21, 29, 31). High school students engaged in a variety of activities, including a semester-long internship program with mentorship and research (28), a 90-day neuroscience health education course (32), a regional brain competition (23), and laboratory research at local institutions (16). The measurement of program outcomes for high school students involved assessments that examined content knowledge and beliefs related to research (16, 17, 25, 30, 32), along with course evaluations and reflections (15, 23, 28). It is evident that a more standardized assessment of neuroscience education and outreach programs, encompassing all age levels, would underscore the significance of such initiatives within the broader neurology community. Furthermore, it could provide insights into the most effective program types and delivery methods for cultivating interest in neuroscience and reinforcing the pipeline toward careers in this field. Additionally, all of the programs in this review only measured and reported on the acute or short-term benefits of the program, failing to evaluate long-term outcomes such as the choice to major in neuroscience as an undergraduate or to pursue a neurology career. The elementary school programs were more experiential in assessing how the programs change based on the target audience’s age. Also, they tended to incorporate more social learning or learning through observation. Middle school programs managed to continue to draw on these classical learning theories. The high school programs tended to be longer and more involved, and many included research opportunities that began to incorporate more problem-based learning and self-directed learning tools, which prepares students for more adult-directed learning theories. Our review showed an equal number of programs reaching elementary, middle, and high school historically marginalized students, indicating that neuroscientists and neurologists recognize the need for strengthening the pipeline for minority students at all age levels. Most programs intended for historically marginalized students had more extended formatting over a few weeks, showing that engaging historically marginalized students in neuroscience over time is more beneficial than shorter programs (11–16). However, a clear need for expansion of these programs also emerges so that the diversity of those in neurology and related fields reflects the diversity of the US population. Only a quarter of programs considered race, ethnicity, school performance, and socioeconomic background when choosing a program audience (11–16). This is problematic when considering earlier literature citing lack of early exposure as a major deterrent for African American medical students to pursue neurology (5). Finally, only two of the programs explicitly focused on mentoring students (11, 28), while 14 others facilitated some short-term interaction with scientists and older students (11–18, 21–24, 26, 28–30). Given the importance of mentorship and positive interactions with neuroscientists in the decision to pursue a career in neurology, more programs could benefit from including a formalized mentoring component (5).

Overall, across all programs, students showed increased neuroscience knowledge, positive attitudes toward neuroscience, education, and STEM careers, and interest in furthering their understanding of the brain. While program delivery and implementation varied, their successes are a testament to the utility of neuroscience and neurology outreach and education programs for all ages and the need to invest in such programs.

### 4.1. Strengths

Our systematic review touched upon a broad range of programs benefiting K-12 students, giving us confidence that researchers and educators recognize the importance of early exposure to neuroscience and neurology for young students. This unique emphasis adds considerable value to our study, positioning it as a foundational reference for future research and advancements in the field. Our research also offers an invaluable resource for stakeholders interested in enhancing K-12 neurology education initiatives. It provides a detailed analysis of program content, duration, efficacy assessment methods, and inclusivity, with particular attention to historically marginalized groups. By emphasizing these aspects, our study contributes to the ongoing development and improvement of K-12 neurology education and outreach efforts, underscoring the significance of continued investment in this critical area.

### 4.2. Limitations

Although all 23 programs successfully implemented an effective neuroscience education and outreach program, there may be publication bias as only successful programs were written about and/or accepted for publication. Another source of publication bias is the underreporting of outreach programs that are not affiliated with academic institutions or are facilitated informally, such as the programs facilitated solely by teachers, individual schools, medical institutions, and community-based organizations. Additionally, given that all 23 programs measured whether they met the intended goals of their intervention and found a positive result, it is possible that to be considered for publication, only programs where efficacy was measured were considered for publication. The programs we reviewed were not designed to assess the long-term effectiveness of their respective neuroscience education and outreach programs, precluding us from determining the effect of such programs on neurology and neuroscience workforces. Another limitation is that we did not include language to search for programs geared toward historically underrepresented groups in our search criteria which may have led to a negative selection bias.

It is also important to note that while our search was conducted about a year ago, the publication range of this systematic review ended over 2 years ago and is not inclusive of some of the more recent initiatives geared toward increasing funding of K-12 neurology and/or neuroscience outreach. For instance, the Science Education Partnership Award (SEPA) under the National Institutes of Health’s (NIH) Research Education Program (R25) sponsors classroom-based projects for Pre-k through 12 students as well as informal science education projects outside the classroom (35). In 2022 through 2023, SEPA funded The Brain Explorer Academy (BEA) at the University of California Irvine, in which high school students from Tier 1 schools engage in a multi-year informal neuroscience education program to foster interest in STEM careers in diverse populations of youth (36). Additionally, SEPA is funding a project entitled “Semilla – planting the seeds of change for Puerto Rico” in which children 9–12 years of age undergo a curriculum to increase awareness about neurobehavioral consequences of toxic stress and disseminate that information with their communities (37). Finally, NeuroLab at the Coastal Marine Biolabs in California is funded through 2024, high school students participate in immersive residential research experiences in developmental neuroscience and genomics, exploring models of nervous system hardwiring and visualizing neurons during embryonic development (38). Thus, there is evidence that the NIH is actively dedicated to funding K-12 programming in the years after those covered by this review.

Finally, this review did not capture any programs that introduce students to neuroscience by way of increasing overall accessibility to the STEM field. For instance, the Journal of Emerging Investigators publishes research in science that is produced by middle and high school students under mentorship (39). While this is broadly a STEM program, it serves some students as a neuroscience outreach program as well, depending on the type of mentorship they are receiving and the research they are conducting. So, K-12 students may be exposed to neuroscience by way of general STEM outreach programs, which was not examined in this review.

### 4.3. Future directions

There needs to be more neuroscience and neurology education and outreach programs with broader geographical reach. Collaboration and support from prominent organizations, such as the AAN, the NIH, the ANA, and the Society for Neuroscience, will likely be critical to continuing this expansion. For example, the AAN sponsors a Neuroscience Research Prize awarded to high school students who conduct neuroscience research and demonstrate potential for future contributions to neuroscience (40). Furthermore, asynchronous resources for K-12 teachers to introduce students to neuroscience and neurology are featured on the AAN website (41). The AAN also addresses the need to expand the neurology pipeline through a dedicated Pipeline Subcommittee, which develops recommendations for engaging 7–12th grade, undergraduate, graduate, and medical students (42). The NIH BRAIN Initiative has developed a web-based, interactive educational experience for high school students to investigate brain mechanisms (43). The NIH funds grants for Principal Investigators who engage high school students in work related to Alzheimer’s disease and Dementia, with a particular interest in diverse groups of students (44).

Large organizations allow K-12 programs to inspire students to become interested in the field by awarding monetary compensation to young researchers who engage with these resources. Yet, these programs are not necessarily accessible to an average high school student who is not aware of the role of the AAN and NIH, and even as researchers, we had to make significant efforts to navigate the organizations’ websites to locate them. To expand programs in organizations such as the AAN and NIH, education committee members should look to partner directly with schools, which has proven effective in the programs from our review. In addition, digital resources such as the NIH BRAIN initiative can engage in social media campaigns to reach the K-12 audience and create adaptable content for elementary and middle school students. Given the need for remote learning due to the COVID-19 pandemic, there is potential for the development of entirely remote neuroscience and neurology education and outreach programs. Future research may examine how the success of remote programs would compare to the type of in-person programs analyzed in this review.

The themes that emerged while conducting our systematic review point to future work on this topic. There has been growing attention in STEM and neuroscience to the mentorship of K-12 students, and while many programs in the review allowed for interaction between K-12 and neuroscientists, few included this as a formal component in the program. Recent efforts such as the NEURON Initiative at the University of Arizona illustrate that formal peer mentoring between high school students and undergraduates is valuable to both groups (45). Three of the studies in our analysis targeted mixed-age groups of undergraduates and K-12 students, although we focused only on the impact on the K-12 students. Future work might focus on the mutually beneficial relationship between undergraduate and K-12 students. In addition, future research could examine the effect that serving as a mentor for K-12 students has on medical and undergraduate students in neuroscience and neurology. Finally, as we restricted our search to only programs that had been implemented, it could be beneficial to look at lesson plans and theorized programs and how they differ from those that had been implemented.

## Data availability statement

The original contributions presented in the study are included in the article/Supplementary materials, further inquiries can be directed to the corresponding author(s).

## Author contributions

MM: Conceptualizatiown, Data curation, Formal analysis, Investigation, Methodology, Project administration, Resources, Supervision, Validation, Writing – original draft, Writing – review & editing. NL: Conceptualization, Data curation, Formal analysis, Investigation, Methodology, Writing – original draft, Writing – review & editing. JE: Conceptualization, Data curation, Formal analysis, Investigation, Methodology, Writing – original draft, Writing – review & editing. KO: Data curation, Formal analysis, Investigation, Writing – original draft, Writing – review & editing. IY: Data curation, Formal analysis, Investigation, Writing – original draft, Writing – review & editing. AK: Data curation, Formal analysis, Investigation, Writing – original draft, Writing – review & editing. RA: Data curation, Formal analysis, Investigation, Writing – original draft, Writing – review & editing. CP: Conceptualization, Data curation, Formal analysis, Investigation, Methodology, Project administration, Resources, Software, Supervision, Validation, Writing – review & editing.
