# Supplementary material for: A critical systematic review of K-12 neurology/neuroscience pipeline programs
Source: Front Med (Lausanne). 2023 Dec 11;10:1281578. doi: 10.3389/fmed.2023.1281578 (PMC10750406; doi:10.3389/fmed.2023.1281578)
Supplement: Supplementary file 1 [file Data_Sheet_1.docx]

| **Appendix A**. Search Strategies | |
| --- | --- |
| PubMed  1070 Results – 7/5/2022 | (Neurology[Mesh] OR Neurology[tiab] OR Neurosciences[Mesh] OR Neuroscience[tiab] OR Neurosciences[tiab]) AND ("primary school"[tiab] OR "elementary school"[tiab] OR "middle school"[tiab] OR "grammar school"[tiab] OR "secondary school"[tiab] OR "high school"[tiab] OR college[tiab] OR undergraduate[tiab] OR Universities[Mesh]) AND (Neurology/education[Mesh] OR Neurosciences/education[Mesh] OR pipeline[tiab] OR "education pipeline"[tiab] OR "career pipeline"[tiab] OR "pipeline program"[tiab] OR "summer program"[tiab] OR Career Choice[Mesh] OR Career[tiab] OR Education[Mesh] OR Education[tiab] OR Learning[Mesh] OR Learning[tiab] OR Motivation[Mesh] OR Motivation[tiab] OR outreach[tiab] OR Curriculum[Mesh] OR Curriculum[tiab] OR Curricula[tiab] OR extracurricular[tiab]) |
| EMBASE  902 results – 7/5/2022 | (*neurology/ or *neuroscience/ or (neurology or neuroscience or neurosciences).ti,ab.) and (*primary school/ or *middle school/ or *high school/ or *college/ or *undergraduate education/ or *university/ or ("primary school" or "elementary school" or "middle school" or "grammar school" or "secondary school" or "high school" or college or undergraduate).ti,ab.) and (*career/ or *education/ or *learning/ or *motivation/ or *curriculum/ or (pipeline or "education pipeline" or "career pipeline" or "pipeline program" or "summer program" or "career choice" or career or education or learning or motivation or outreach or curriculum or curricula or extracurricular).ti,ab.) |
| PsycINFO  502 results – 7/5/2022 | (*neurology/ or *neurosciences/ or (neurology or neuroscience or neurosciences).ti,ab.) and (*Elementary Schools/ or *Middle Schools/ or *Secondary Education/ or *High Schools/ or *Colleges/ or ("primary school" or "elementary school" or "grammar school" or "secondary school" or "high school" or college or undergraduate).ti,ab.) and (*Occupational Choice/ or *Occupations/ or *Education/ or *Learning/ or *Motivation/ or *Curriculum/ or *Educational Programs/ or (pipeline or "educational pipeline" or "career pipeline" or "pipeline program" or "summer program" or "career choice" or career or education or learning or outreach or curriculum or curricula or extracurricular).ti,ab.) |
| Education Source + ERIC  418 results – 7/5/2022  378 with duplicates removed | (DE "Neurology" OR TI neurology OR AB neurology OR DE "Neurosciences" OR TI neuroscience OR AB neuroscience OR TI neurosciences OR AB neurosciences) AND (DE "Primary schools" OR TI "primary school" OR AB "primary school" OR DE "Elementary Schools" OR TI "elementary school" OR AB "elementary school" OR DE "Middle schools" OR TI "middle school" OR AB "middle school" OR TI "grammar school" OR AB "grammar school" OR DE "Secondary schools" OR TI "secondary school" OR AB "secondary school" OR DE "High schools" OR TI "high school" OR AB "high school" OR DE "Universities & colleges" OR TI "college"  OR AB "college" OR DE "Undergraduates" OR TI undergraduate OR AB undergraduate) AND (DE "Neurology education" OR TI pipeline OR AB pipeline OR TI "education pipeline" OR AB "education pipeline" OR TI "career pipeline" OR AB "career pipeline" OR TI "pipeline program" OR AB "pipeline program" OR TI "summer program" OR AB "summer program" OR DE "Summer Programs" OR DE "Career Choice" OR TI "career choice" OR AB "career choice" OR DE "Occupational Aspiration" OR TI career OR AB career OR DE "Education" OR TI education OR AB education OR DE "Learning" OR TI learning OR AB learning OR DE "Motivation" OR TI Motivation OR AB Motivation OR TI outreach OR AB outreach OR DE "Curriculum" OR TI Curriculum OR AB Curriculum OR TI Curricula OR AB Curricula OR TI extracurricular OR AB extracurricular) |
